# Interprofessional education in medical schools in Japan

**DOI:** 10.1371/journal.pone.0210912

**Published:** 2019-01-17

**Authors:** Takami Maeno, Junji Haruta, Ayumi Takayashiki, Hisashi Yoshimoto, Ryohei Goto, Tetsuhiro Maeno

**Affiliations:** Faculty of Medicine, University of Tsukuba, Tsukuba, Ibaraki, Japan; Oakland University, UNITED STATES

## Abstract

Interprofessional education (IPE) for medical students is becoming increasingly important, as reflected in the increasing number of medical schools adopting IPE. However, the current status of and barriers to pre-registration IPE implementation in Japanese medical schools remain unknown. The purpose of this study was to clarify the status and barriers of IPE implementation in medical schools in Japan. We conducted a curriculum survey from September to December 2016 of all 81 medical schools in Japan. We mailed the questionnaire and asked the schools’ undergraduate education staff to respond. The survey items were the IPE implementation status and barriers to program implementation. Sixty-four of the 81 schools responded (response rate 79.0%), of which 46 (71.9%) had implemented IPE, 42 (89.1%) as compulsory programs. Half of IPE programs were implemented in the first 2 years, while less than 10% were implemented in the latter years of medical programs. As part of the IPE programs, medical students collaborated with a wide range of professional student groups. The most common learning strategy was lectures. However, one-third of IPE programs used didactic lectures without interaction between multi-professional students. The most common perceived major barrier to implementing IPE was adjustment of the academic calendar and schedule (82.8%), followed by insufficient staff numbers (73.4%). Our findings indicate that IPE is being promoted in undergraduate education at medical schools in Japan. IPE programs differed according to the circumstances of each school. Barriers to IPE may be resolved by improving learning methods, introducing group discussions between multi-professional students in lectures or introducing IPE programs using team-based learning. In summary, we demonstrated the current status and barriers of IPE implementation in Japanese medical schools. Our findings will likely lead to the promotion of IPE programs in Japan.

## Introduction

The increasing complexity of medical systems in our aging society has led to increased emphasis on a patient-centered collaborative approach to care [1]. To improve collaborations, interprofessional education (IPE) is an essential strategy in both pre-licensure and post-licensure contexts. IPE has been promoted as an integral part of undergraduate education. In a survey of pre-registration interprofessional education in the United Kingdom (UK) in 2010–2012, 52 of 127 educational institutions responded with information concerning 63 IPE courses and modules [2]. In Australia and New Zealand, a survey targeting 43 universities offering nursing, pharmacy and medical programs received responses from 31 of the 43 target universities, 80% of which reported implementing IPE [3].

At the postgraduate level, interprofessional work (IPW) is important for physicians working in healthcare teams. Previous studies have reported that interprofessional hierarchies have considerable bearing on communication and collaboration [4]. Without undergraduate IPE, physicians might harbor the belief that they make decisions in a top-down approach within medical teams. Such thinking can be a barrier to IPW in the field. However, while many physicians tend to adopt a negative attitude towards IPW [5–7], studies targeting medical students report that students who have received IPE tend to have a positive attitude towards IPW [8,9]. This evidence indicates that it is important to implement IPE to medical students at the undergraduate level. “Outcomes for graduates,” a publication of the General Medical Council [10] which describes the outcomes of and sets standards for undergraduate medical education in the UK, has set “learn and work effectively within a multi-professional team” as a learning goal for medical professionals.

A survey on IPE for medical students targeting 126 US medical schools obtained responses from 48 schools, 66% of which reported having implemented IPE [11]. Another survey targeting 17 Canadian medical schools obtained responses from 12 schools, all of which offered mandatory IPE programs [12]. In Japan, a team approach to health care has also been included in the model core curriculum for undergraduate medical education [13], which describes the outcomes of and sets standards for undergraduate medical education in Japan. Until two decades ago, however, no university or college in Japan had incorporated a large-scale IPE program into its educational curriculum to facilitate the collaborative learning process of multi-professional students. In contrast, in the past 20 years, there has been an upsurge of interest in IPE from academic institutions, and the number of colleges that have introduced IPE into their curriculum since 2000 has increased [14].

Two previous studies reported that IPE implementation in medical schools in Japan ranged from only 34.8% [15] to 37.5% [16]. Response rates to these studies were low, however, at 32.5% and 10.0%, respectively. These studies also targeted a wide range of medical and healthcare and social professions. Studies have also reported many obstacles to implementing IPE, such as schedule adjustment, funding limitations, and shortage of human resources, among others [11,12,15]. Implementation is also hampered by the rapidly changing medical education curriculum [17]. Therefore, the current rate of pre-registration IPE implementation in Japanese medical schools remains unknown. Clarification of the current status and barriers of IPE programs will improve the process of introducing IPE and resolve barriers to IPE, and will lead to the promotion of IPE in Japan.

Here, we aimed to clarify the current rate of IPE implementation specifically in medical schools in Japan, and the barriers associated with implementation.

## Methods

We conducted a national curriculum survey from September to December 2016 of all 81 medical schools in Japan. We mailed a questionnaire to each medical school’s teaching affairs office and asked the undergraduate education staff to respond. We sent reminder postcards and made a final phone call to the medical schools that did not respond.

We explained the purpose and methods of the research in written documents and regarded the return of completed survey forms as consent to participate in the research. This study was approved by the Ethics Committee of the University of Tsukuba (No. 1091).

### Definition of IPE

In this study, we defined IPE as a program in which medical students and students from other departments (different professional groups) learn together.

### Survey items

Given that there was no standardized questionnaire available to address the aims of the study [15], we developed a questionnaire based on previous research [2,11,15]. We reviewed large-scale surveys conducted in the UK, US and Japan. These instruments were not unified because the contents of the surveys differed depending on the culture and context of the country and institution. We developed a questionnaire consisting of general items used in previous studies that investigated IPE curricula. The questionnaire was not pretested for validity or reliability prior to administration in the study.

The survey items were implementation status of IPE (compulsory/elective, students’ year level at implementation, number of hours, the professional student groups learning with medical students, learning strategy, evaluation method, and presence or absence of university cooperation), implementation of faculty development (FD) in IPE, and barriers to implementing an IPE program. Respondents were asked to rate the likelihood that a factor was a barrier on a five-point Likert-type scale: “5 = major barrier”, “4 = somewhat”, “3 = neutral”, “2 = not so much”, “1 = no barrier” (S1–S4 Appendices).

The number of IPE programs was defined as the total number of IPE programs per school, which was equivalent to the number of IPE programs offered for medical students during the six-year undergraduate medical program.

### Analysis

We regarded responses of 4 and 5 as indicating that the school perceived the factor as a barrier to IPE, and therefore combined responses 4 and 5. Descriptive statistics were computed. Categorical data are presented as number and percentage. The analyses were performed using IBM SPSS Statistics Version 22.0.

## Results

Sixty-four of the 81 medical schools in Japan responded to the questionnaire, with a response rate of 79.0%. The staff at each of the medical schools provided responses. Of the medical schools that responded, 32 were national (76.2% of all national schools), 8 were prefectural public (100.0% of all prefectural public schools), and 30 were private (76.7% of all private schools) schools (Table 1). Forty-six of the 64 (71.9%) medical schools had implemented a total of 111 IPE programs.

**Table 1:** Characteristics of participating medical schools (n = 64).

		n	%
Ownership			
	National	32	50.0
	Prefectural public	8	12.5
	Private	23	35.9
	Other	1	1.6
IPE implementation			
	Yes	46	71.9
	No	18	28.1

IPE: interprofessional education

### Medical schools that implemented IPE

Table 2 summarizes the characteristics of the 46 medical schools that implemented IPE programs. Forty-one schools (90%) had compulsory IPE programs. Seventeen schools (40%) conducted only one IPE program, while 29 schools (60%) implemented two or more IPE programs. Some schools repeated IPE programs depending on the students’ year level, for example, using a spiral curriculum. Fourteen schools (30%) implemented faculty development (FD) in IPE, and 18 schools (40%) introduced collaboration among schools.

**Table 2:** Characteristics of medical schools that have implemented IPE (n = 46).

		n	%
Compulsory/elective			
	Compulsory	41	89.1
	Elective	5	10.9
Number of IPE programs*			
	1	17	37.0
	2	11	23.9
	3	11	23.9
	4	3	6.5
	5 or more	4	8.7
Faculty development implementation□		14	30.4
Collaboration among universities		18	39.1

*The number of IPE programs is the total number of IPE programs per school.

### IPE programs

The 46 schools that implemented IPE programs provided responses regarding 111 programs. Table 3 summarizes the characteristics of the 111 IPE programs.

**Table 3:** Characteristics of IPE programs (n = 111).

Characteristic		n	(%)
Student year level at program implementation	1st	46	(41.4)
	2nd	12	(10.8)
	3rd	14	(12.6)
	4th	17	(15.3)
	5th	10	(9.0)
	6th	3	(2.7)
	Multiple years	9	(8.1)
Professional student groups learning with medical students (multiple answers allowed)	Nursing	88	(79.3)
	Pharmacy	52	(46.8)
	Physical therapy	29	(26.1)
	Occupational therapy	28	(25.2)
	Medical technology	17	(15.3)
	Dental	14	(12.6)
	Radiological technology	10	(9.0)
	Dietician/registered dietitian	9	(8.1)
	Social worker	7	(6.3)
Learning strategy (multiple answers allowed)	Lectures with interaction between multi-professional students	54	(48.6)
	Lectures without interaction between multi-professional students	41	(36.9)
	Group discussion	64	(57.7)
	Team-based learning	24	(21.6)
	Problem-based learning	18	(16.2)
	Simulation	16	(14.4)
	Practical training at a health care and welfare site with interaction between multi-professional students	23	(20.7)
	Practical training at a health care and welfare site without interaction between multi-professional students	8	(7.2)
	e-learning	6	(5.4)
Assessment	Conducted	92	(82.9)
Assessment methods (multiple answers allowed)	Attendance	85	(76.6)
	Report	75	(67.6)
	Observation	41	(36.9)
	Test	22	(19.8)
	Portfolio	7	(6.3)

Medical students in Japan receive medical education for 6 years, which in general comprises general liberal arts education for the first 2 years, lectures on basic medicine followed by clinical practice in the next 2 years, and clinical practice training in the last 2 years. Half of IPE programs were implemented in the first 2 years, while less than 10% of programs were implemented in the last 2 years, indicating that relatively few programs were implemented in the latter years of medical programs.

Among the professional student groups with whom medical students collaborated, nursing students were the most common (79.3%), followed by pharmacy (46.8%), physical therapy (26.1%), and occupational therapy students (25.2%). The programs also included collaboration with other types of professional student groups, such as medical engineers and orthoptists, with some also including collaboration with professional groups outside of the medical field such as nursery teachers.

The most common learning strategies used in IPE programs were group discussion and lectures. Of all 111 IPE programs, 48.6% and 36.9% used lectures that did or did not incorporate interaction among professional student groups, respectively. Meanwhile, 21.6% and 16.2% of IPE programs used team-based learning (TBL) and problem-based learning (PBL) strategies, respectively. Twenty-three medical schools introduced practical training at a health care and welfare site. Of all 111 IPE programs, 20.7% and 7.2% used practical training at a health care and welfare site that did and did not incorporate interaction between multi-professional students, respectively. Most of these were conducted among lower grade students for early exposure, while only 9 schools implemented practical training at a health care and welfare site for students in the fourth year or above. Furthermore, only 4 schools implemented compulsory practical training at a health care and welfare site.

Of the 111 IPE programs, 92 (82.9%) conducted assessments in their IPE programs. The most common assessment method was attendance (76.6%), followed by report (67.6%). Some programs assessed progress in the IPE programs by observations (19.8%) and portfolios (6.3%).

### Perceived barriers to implementing IPE

The most common perceived major barrier to implementing IPE was adjustment of the academic calendar and schedule (82.8%), followed by insufficient staff numbers (73.4%) and lack of classroom space (51.6%) (Table 4). More than half of medical schools perceived these factors as barriers. About 40% of schools cited funding limitations and insufficient understanding of educational methods by staff as barriers.

**Table 4:** Perceived barriers to implementing IPE at Japanese medical schools (n = 64).

Factor	n	(%)
Adjustment of academic calendar and schedule	53	(82.8)
Insufficient staff numbers	47	(73.4)
Lack of classroom space	33	(51.6)
Funding limitations	25	(39.1)
Insufficient understanding of educational methods by staff	24	(37.5)
Difficulty developing teaching materials	23	(35.9)
Lack of institutional understanding	18	(28.1)
Difficulty finding other disciplines for collaboration	18	(28.1)

Respondents were asked to score perceived barriers to implementing IPE on a five-point Likert-type scale (“5 = major barrier”, “4 = somewhat”, “3 = neutral”, “2 = not so much”, “1 = no barrier”). We regarded responses of 4 and 5 as indicating that the school perceived the factor as a barrier to IPE; therefore, 4 and 5 were combined.

## Discussion

This survey found that IPE has been adopted by approximately 70% of medical schools in Japan, with 90% of these schools implementing IPE as a compulsory program. The IPE programs were implemented according to the actual circumstances of each school. Clarification of the current status of IPE programs is expected to improve the process of introducing IPE and resolve barriers to IPE, and will lead to the promotion of IPE in Japan.

The survey yielded a high response rate of 79.0%. Response rates in previous studies were low [15, 16]. Previous studies targeted a wide range of medical and healthcare and social professions, and did not have strategies for reminding schools that had not responded to their questionnaires. In this study, we focused on IPE implementation in medical schools. We sent reminder postcards and made phone calls to the schools that had not responded to our questionnaire. The high response rate was therefore achieved by improving on previous research methods. Therefore, our results are likely generalizable to all Japanese medical schools.

IPE is being promoted in undergraduate education at medical schools in Japan. Surveys on pre-registration IPE programs for medical students in Japan conducted in 2012 [15] and 2013 [16] showed that 34.8% and 37.5% of Japanese medical schools implemented IPE, respectively. Although the settings of these studies differed from those of the present study, our findings suggest that implementation of IPE in Japan is rapidly increasing. IPE is becoming increasingly important worldwide, and is increasingly being adopted in medical schools in Japan. The curricula and resources differed among medical schools, suggesting that each IPE program was implemented according to the actual circumstances of each school. Sixty percent of the medical schools implemented two or more IPE programs. An interprofessional spiral curriculum model has been used to implement IPE from the early years of medical programs, to repeatedly expose students to collaboration throughout their training [18]. We found that some medical schools repeated the IPE program according to the students’ level using a spiral curriculum.

Lectures were the most common learning strategy adopted for IPE. A previous Japanese study reported that approximately half of IPE programs were delivered through lectures [16]. However, in our study, one-third of IPE programs used didactic lectures without interaction between multi-professional students. IPE is most often defined as an “occasion when two or more professions learn with, from and about each other to improve collaboration and the quality of care” [19]. Therefore, in cases where students of various professions attend the same lecture, the education does not meet the definition of IPE unless there is interaction between the multi-professional students.

The biggest barrier to implementing IPE was schedule adjustment among school departments. The very busy and strict curricula of individual departments make it difficult to identify mutually free time to implement an IPE program. Common lectures combining students from different departments might therefore be valuable opportunities for implementing IPE by introducing group discussions between multi-professional students. TBL is one approach for adapting lectures for this purpose. TBL has the advantage that a large class can be run by a small number of staff members. TBL also offers a number of other advantages, such as collaboration and active participation by learners in the education process, which are essential in team medical care [20,21]. Owing to its effectiveness, TBL has spread rapidly in medical education in recent years [22,23], and its effectiveness in IPE has been reported [24]. While PBL is also a useful strategy for IPE [25], it is associated with difficulties with staffing a large number of teachers as tutors, is time consuming and requires schedule adjustments between departments. Our study results showed that only 16.2% of IPE programs used PBL. TBL may therefore be an effective learning strategy for IPE.

Some medical schools adopted practical training at a health care welfare site, although such practical training was limited in the latter years of medical programs. This may be because medical students receive medical education for 6 years in Japan, which is longer than students from other departments. This difference in education period may underlie the mismatch in IPE curricula between medical students and those from other departments. Alternatively, it may be difficult for university hospitals, where medical students primarily conduct clinical practice, to implement interprofessional practical training. However, a previous study suggested that practical training in primary care settings may be an opportunity for IPE, and that integration of IPE into community-based learning might be an effective strategy [26].

Factors such as schedule adjustment among different departments and insufficient staff numbers were perceived as major obstacles to implementing IPE in Japanese medical schools, as was reported in previous research [11,12,15]. Although there are restrictions, such as those mentioned above, these barriers may be resolved by introducing group discussion among multi-professional student groups in lectures and introducing TBL.

Thirty percent of schools conducted FD for IPE. Facilitation of faculty staff is important for effective IPE [27]. However, as mentioned above, IPE is relatively new in Japan, and most teaching staff have no experience with either receiving or teaching IPE. Therefore, FD is very important for informing staff of the purpose and educational methods of IPE.

This study has several limitations. First, there may have been a non-responder bias. The schools that did not participate in the survey may not have implemented IPE. The actual rate of IPE implementation may therefore be lower than that reported in this study. Second, there is a possibility that the survey respondents do not properly understand IPE. For example, some schools indicated that they conducted lectures and practical training at a health care and welfare site without interaction between multi-professional students as IPE. As mentioned above, the lack of interaction between multi-professional students fails to meet the definition of IPE. Therefore, the actual IPE implementation rate may be lower. Similarly, a previous survey of pre-registration IPE conducted in Australia and New Zealand reported that 80% of target universities answered that they offered IPE programs, but only 24% of these programs met the accepted definition of IPE [3]. Therefore, strategies are needed to enhance understanding of IPE. Third, the questionnaire was not pretested for validity or reliability prior to administration in the study. We developed this questionnaire because there were no standardized questionnaires to address the aims of the study. Future studies should examine the validity and reliability of the questionnaire. Additionally, all data were obtained using only the questionnaire. We did not check published information or the schools’ webpages for the contents of the curricula. Therefore, the actual status of each program is unknown. To obtain more accurate information on the curricula, future studies should gather additional information such as that from published information or the schools’ webpages, or conduct an interview survey. Onishi et al. reported that learning experiences in undergraduate education were significantly associated with higher collaborative practice scores in physicians [9]. Although this study reported a relatively high implementation rate for IPE in Japan, reports examining the effects of undergraduate IPE in Asia are limited [28]. Therefore, further research is needed.

## Conclusions

IPE is being promoted in undergraduate education at medical schools in Japan. IPE programs differed according to the circumstances of each medical school. Barriers to IPE may be resolved by improving learning methods, introducing group discussions between multi-professional students in lectures and introducing IPE programs using TBL. Clarification of the current status and barriers of IPE in Japanese medical schools may help to promote IPE programs in Japan. IPE is a crucial component of undergraduate medical curricula around the world. The results of this study will also be useful in Asian countries that are developing IPE.

## Supporting information

S1 Appendix Survey questionnaire in English. (DOCX)

S2 Appendix Question 2 attachment in English. (DOCX)

S3 Appendix Survey questionnaire in Japanese. (DOCX)

S4 Appendix Question 2 attachment in Japanese. (DOCX)
