# Supplementary material for: Interprofessional education in medical schools in Japan
Source: PLoS One. 2019 Jan 17;14(1):e0210912. doi: 10.1371/journal.pone.0210912 (PMC6336262; doi:10.1371/journal.pone.0210912)
Supplement: S1 Appendix — (DOCX) [file pone.0210912.s001.docx]

**National curriculum survey of**

**pre-registration interprofessional education for medical schools**

Please read first

In this survey, we defined interprofessional education (IPE) as a program in which medical students and students from other departments (different professional groups) learn together.

Please describe the status of implementation of IPE at your school.

1. Does your school implement IPE programs for medical students in which medical students and students from other departments (different professional groups) learn together?

（　　　）① Yes → Question 2

（　　　）② No → Question 3

2. To IPE-implementing schools, please complete the following table on the educational contents of each grade. Please also complete the attachment on the specific contents. After completing this,

go to Question 4.

| No. | Students’ year level at implementation | Subject name | Compulsory/elective |
| --- | --- | --- | --- |
| Example | 2 | interprofessional collaborative practice | compulsory　・ elective |
| 1 |  |  | compulsory　・ elective |
| 2 |  |  | compulsory　・ elective |
| 3 |  |  | compulsory　・ elective |
| 4 |  |  | compulsory　・ elective |
| 5 |  |  | compulsory　・ elective |
| 6 |  |  | compulsory　・ elective |
| 7 |  |  | compulsory　・ elective |
| 8 |  |  | compulsory　・ elective |
| 9 |  |  | compulsory　・ elective |

　　　If there is not enough space, please provide your response in any available space on this form or on another sheet of paper.

3. To non-IPE-implementing schools, is your school planning to introduce IPE in the future?

（　　　）① Yes

（　　　）② No

To all universities:

4. Does your school implement Faculty Development related to IPE?

（　　　）① Yes

（　　　）② No

5. Are the following factors obstacles to introducing/implementing an IPE program?

Circle the most appropriate answer for each option.

|  | 1 | 2 | 3 | 4 | 5 | 6 |
| --- | --- | --- | --- | --- | --- | --- |
|  | Major  Barrier | somewhat | neutral | not so  much | no  barrier | do not know  /not applicable |
| (1) Lack of institutional understanding | 1 | 2 | 3 | 4 | 5 | 6 |
| (2) Difficulty finding other disciplines  for collaboration | 1 | 2 | 3 | 4 | 5 | 6 |
| (3) Adjustment of academic calendar and schedule | 1 | 2 | 3 | 4 | 5 | 6 |
| (4) Funding limitations | 1 | 2 | 3 | 4 | 5 | 6 |
| (5) Insufficient staff numbers | 1 | 2 | 3 | 4 | 5 | 6 |
| (6) Lack of classroom space | 1 | 2 | 3 | 4 | 5 | 6 |
| (7) Insufficient understanding of educational methods by staff | 1 | 2 | 3 | 4 | 5 | 6 |
| (8) Difficulty developing teaching materials | 1 | 2 | 3 | 4 | 5 | 6 |

(9) Other. Please provide details

Thank you for your cooperation.
