# Supplementary material for: Interprofessional education in medical schools in Japan
Source: PLoS One. 2019 Jan 17;14(1):e0210912. doi: 10.1371/journal.pone.0210912 (PMC6336262; doi:10.1371/journal.pone.0210912)
Supplement: S2 Appendix — (DOCX) [file pone.0210912.s002.docx]

**Question 2** **Attachment**

| **Subject name** | No （　　　　） |
| --- | --- |
| **Number of hours** |  |
| **Professional student groups learning with medical**  **students**  Please circle all that apply | Nursing 　・　 Pharmacy 　・　 Physical therapy 　・　 Occupational therapy 　・  Speech-Language-Hearing Therapist ・ Medical technology ・ Radiological technology Dietician 　・　 Registered dietitian 　・　 Social worker 　・　 Care worker ・  Psychiatric social worker ・ Dental  Other （　　　　　　　　　　　　　　　　　　　　　　　　　　　　　　　　　　　　　　　　　　　　　　　　　　　　　　　　） |
| **Collaboration among universities** | Yes　・　No |
| **Goal setting** | Yes　・　No |
| **Learning strategy** | 1-1　 Lectures without interaction between multi-professional students |
| Please circle all that apply | 1-2　 Lectures with interaction between multi-professional students |
|  | 2 Group discussion |
|  | 3　 Problem-based learning： PBL |
|  | 4　Team-based learning： TBL |
|  | 5 Simulation (program using simulated patients and/or simulators） |
|  | 6-1 Practical training at a health care and welfare site without interaction between multi-professional students |
|  | 6-2 Practical training at a health care and welfare site with interaction between multi-professional students |
|  | 7　E-Learning |
|  | 8　 Other （　　　　　　　　　　　　　　　　　　　　　　　　　　　　　　　　　　　　　　　　　　　　　　　　　　　　　　　　　　） |
| **Assessment** | Yes　・　No 　→　 If yes, please indicate the assessment method(s) below |
| Please circle all that apply | Attendance ・ Test　・　 Report　・　 Observation　・  Interview with a simulated patient and/or simulation ・　Portfolio  Other （　　　　　　　　　　　　　　　　　　　　　　　　　　　　　　　　　　　　　　　　　　　　　　） |
| **Other** |  |
| Please add special notes such as features of the program if applicable. |  |

※If possible please attach a copy of the syllabus.
