# Supplementary material for: Interprofessional education in medical schools in Japan
Source: PLoS One. 2019 Jan 17;14(1):e0210912. doi: 10.1371/journal.pone.0210912 (PMC6336262; doi:10.1371/journal.pone.0210912)
Supplement: S3 Appendix — (DOCX) [file pone.0210912.s003.docx]

**医学部医学科における卒前専門職連携教育に関する全国調査**

はじめに

**本調査における「専門職連携教育」は、**

**医学生と他の学科（異なる職種）の学生が共に学ぶものです。**

貴学における専門職連携教育の実施状況についてお答えください。

１．貴学では、医学生と他職種の学生が共に学ぶ専門職連携教育を行っていますか。

（　　　）① はい → 設問2へ

（　　　）② いいえ → 設問3へ

２. 専門職連携教育を行っている大学におうかがいします。各学年の教育内容について

以下の表にご記入の上、具体的な内容について色のついた別紙にご記入ください。ご記入後、

設問４にお進みください。

| No | 医学生の 学年 | 科目名 | 必修/選択 |
| --- | --- | --- | --- |
| 例 | 2 | インタープロフェッショナル演習 | 必修　・選択 |
| 1 |  |  | 必修　・選択 |
| 2 |  |  | 必修　・選択 |
| 3 |  |  | 必修　・選択 |
| 4 |  |  | 必修　・選択 |
| 5 |  |  | 必修　・選択 |
| 6 |  |  | 必修　・選択 |
| 7 |  |  | 必修　・選択 |
| 8 |  |  | 必修　・選択 |
| 9 |  |  | 必修　・選択 |

　　　欄が足りない場合、同様の項目について、この用紙のあいている箇所又は別の用紙にご記入ください。

３. 専門職連携教育を行っていない大学におうかがいします。今後、専門職連携教育を実施する予定はありますか。

（　　　）① はい

（　　　）② いいえ

以下の設問は全ての大学におうかがいします。

４. 専門職連携教育に関するFaculty Developmentを行っていますか。

（　　　）① はい

（　　　）② いいえ

５． 専門職連携教育プログラムを導入/実施する上で、以下の要因は障害になっていますか。

あてはまるものに〇をつけてください。

１ ２ ３ ４ ５ ６

非常に やや どちらでも 　　あまり　　まったく　わからない
 ある ある ない ない ない 　　/該当しない

(1)大学の理解が進んでいない　 １ ２ ３ ４ ５ ６

(2)連携する学科をみつけるのが困難 １ ２ ３ ４ ５ ６

(3)学科間のスケジュール調整が困難 １ ２ ３ ４ ５ ６

(4)財源不足 １ ２ ３ ４ ５ ６

(5)教員不足 １ ２ ３ ４ ５ ６

(6)場所・教室がない １ ２ ３ ４ ５ ６

(7)教員が教育方法を理解するのが困難 １ ２ ３ ４ ５ ６

(8)教材開発が困難 １ ２ ３ ４ ５ ６

(9)その他、障害となることがあればご記入ください

アンケートはこれで終了です。ご協力ありがとうございました。
