# Supplementary material for: Interprofessional education in medical schools in Japan
Source: PLoS One. 2019 Jan 17;14(1):e0210912. doi: 10.1371/journal.pone.0210912 (PMC6336262; doi:10.1371/journal.pone.0210912)
Supplement: S4 Appendix — (DOCX) [file pone.0210912.s004.docx]

設問2 別紙

| **科目名** | No（　　　　） |
| --- | --- |
| **時間数** |  |
| **対象職種** | 看護師　・　薬剤師　・　理学療法士　・　作業療法士　・　言語聴覚士 ・ 臨床検査技師 ・ 診療放射線技師 |
| 医学生以外の参加学生の職種について当てはまるものすべてに○ | 栄養士　・ 管理栄養士　・　社会福祉士　・　介護福祉士 ・ 精神保健福祉士 ・ 歯科医師 |
|  | その他 （　　　　　　　　　　　　　　　　　　　　　　　　　　　　　　　　　　　　　　　　　　　　　　　　　　　　　　　　） |
| **他大学との連携** | あり　・　なし |
| **目標設定** | あり　・　なし |
| **学習方法** | 1-1　講義 ： 異なる職種の学生同士の討論やグループワークなどを行っていないもの |
| 当てはまるものすべてに○ | 1-2　講義 ：異なる職種の学生同士の討論やグループワークなどを行っているもの |
|  | 2 グループ討論 |
|  | 3　問題基盤型学習(Problem based learning：PBL) |
|  | 4　チーム基盤型学習（Team-based learning ：TBL） |
|  | 5 シミュレーション（模擬患者やシミュレーターを用いた学習） |
|  | 6-1 保健医療福祉現場の実習：異なる職種の学生同士の討論やグループワークなどを行っていないもの |
|  | 6-2 保健医療福祉現場の実習：異なる職種の学生同士の討論やグループワークなどを行っているもの |
|  | 7　E-Learning |
|  | 8　その他（　　　　　　　　　　　　　　　　　　　　　　　　　　　　　　　　　　　　　　　　　　　　　　　　　　　　　　　　　　） |
| **学生評価** | あり　・　なし　→　学生評価を行っている場合、行っている評価について以下にお答えください |
| 当てはまるものすべてに○ | 出席 ・ 試験　・　レポート　・　グループ学習や実習の観察　・　模擬患者の面接やシミュレーション |
|  | ポートフォリオ ・ その他（　　　　　　　　　　　　　　　　　　　　　　　　　　　　　　　　　　　　　　　　　　　　　　） |
| **その他** |  |
| プログラムの特徴など特記事項があればご記入ください。 |  |

※可能でしたらシラバスのコピーを添付してください。
